# CSXAI: a lightweight 2D CNN-SVM model for detection and classification of various crop diseases with explainable AI visualization

**DOI:** 10.3389/fpls.2024.1412988

**Published:** 2024-07-05

**Authors:** Reazul Hasan Prince, Abdul Al Mamun, Hasibul Islam Peyal, Shafiun Miraz, Md. Nahiduzzaman, Amith Khandakar, Mohamed Arselene Ayari

**Affiliations:** ^1^ Department of Electrical and Computer Engineering, Rajshahi University of Engineering and Technology, Rajshahi, Bangladesh; ^2^ Department of Computer Science and Engineering, Tejgaon College, Dhaka, Bangladesh; ^3^ Department of Computer Science and Engineering, Rajshahi University of Engineering and Technology, Rajshahi, Bangladesh; ^4^ Department of Electrical Engineering, College of Engineering, Qatar University, Doha, Qatar; ^5^ Department of Civil and Architectural Engineering, Qatar University, Doha, Qatar; ^6^ Technology Innovation and Engineering Education Unit, Qatar University, Doha, Qatar

**Keywords:** convolutional neural network (CNN), support vector machine (SVM), gradient-weighted class activation mapping (GRAD-CAM), pre-trained models, plant diseases

## Abstract

Plant diseases significantly impact crop productivity and quality, posing a serious threat to global agriculture. The process of identifying and categorizing these diseases is often time-consuming and prone to errors. This research addresses this issue by employing a convolutional neural network and support vector machine (CNN-SVM) hybrid model to classify diseases in four economically important crops: strawberries, peaches, cherries, and soybeans. The objective is to categorize 10 classes of diseases, with six diseased classes and four healthy classes, for these crops using the deep learning-based CNN-SVM model. Several pre-trained models, including VGG16, VGG19, DenseNet, Inception, MobileNetV2, MobileNet, Xception, and ShuffleNet, were also trained, achieving accuracy ranges from 53.82% to 98.8%. The proposed model, however, achieved an average accuracy of 99.09%. While the proposed model's accuracy is comparable to that of the VGG16 pre-trained model, its significantly lower number of trainable parameters makes it more efficient and distinctive. This research demonstrates the potential of the CNN-SVM model in enhancing the accuracy and efficiency of plant disease classification. The CNN-SVM model was selected over VGG16 and other models due to its superior performance metrics. The proposed model achieved a 99% F1-score, a 99.98% Area Under the Curve (AUC), and a 99% precision value, demonstrating its efficacy. Additionally, class activation maps were generated using the Gradient Weighted Class Activation Mapping (Grad-CAM) technique to provide a visual explanation of the detected diseases. A heatmap was created to highlight the regions requiring classification, further validating the model's accuracy and interpretability.

## 1 Introduction

In Bangladesh, agriculture is crucial due to a growing population and higher food demand. Besides, the gross national income of the country and the families of the farmers depend on the agriculture field. Many countries rely on agricultural products and allied businesses as their primary source of income. One of the most basic and crucial necessities for any country is the safety and security of agricultural products Akbar et al. (2022). As plants are the health of agricultural development, so it is essential to increase the production of crops by ensuring the health of plant leaves. To boost plant yield, it’s essential to address the issue of low yield caused by diseases from bacteria, viruses, and fungi. Moreover, Plant leaf diseases not only impact our daily lives but also have a terrible impact on farmers whose families depend on the production of plants. Identifying and classifying these diseases manually is both time-consuming and prone to errors. To address this, we suggest a deep learning approach for accurate and efficient identification and classification of plant leaf diseases. This method utilizes neural networks to extract characteristics of diseased parts, enhancing the accuracy of disease area classification. Detecting these plant diseases can help prevent them, and deep learning methods are effective for identification because they analyze data directly, focusing on specific task outcomes. This paper outlines the steps in a plant disease detection system and compares deep learning techniques for detecting plant diseases. To identify diseases by applying deep learning techniques, this paper introduces four kinds of crop leaves - Cherry, Peach, Strawberry, and Soybean.

Cherries hold notable importance in human health due to their rich nutritional profile and potential health benefits. Packed with antioxidants, particularly anthocyanins, cherries contribute to combating oxidative stress and inflammation, potentially promoting heart health and reducing the risk of chronic diseases. However, the cultivation of cherries is not without challenges, as various diseases, such as bacterial canker, brown rot, and powdery mildew, can pose significant drawbacks. The cherry leaves infected by Podosphaera pannosa will suffer powdery mildew, which is a serious disease threatening the cherry production industry Zhang et al. (2019). Thus, identifying a cherry leaf infected by Podosphaera pannosa only needs to identify whether the cherry leaf is healthy or diseased. To identify the diseased cherry leaves in the early stage, a combined technique of machine learning and deep learning have been used.

Peaches, both delicious and nutritious, hold significant importance in the realm of nutrition and well-being. Several diseases can attack peaches, including Bacterial spots, also known as Bacteriosis or shot holes. This disease also can be called peach spot. However, Bacteriosis severely affects peach crop production. Bacteriosis typically develops on the peach leaves first; therefore, the leaves are the primary source for recognizing plant disease Ebrahimi et al. (2017). The diseases reduce the yield of peaches and cause harm to human health. Thus, it is important to find rapid and accurate methods to identify peach diseases and further locate and segment the areas of the lesion in earlier stages Yao et al. (2022).

In many parts of the world, soybeans are the main food crop for people and an important source of oil for human consumption. But in recent years, some factors such as natural disasters, soil erosion, and fertilizer unreasonably lead to the occurrence of crop diseases. These diseases seriously affect soybean yield and quality in some aspects Gui et al. (2015). Traditional diagnosis of these diseases relies on disease symptom identification based on naked-eye observation by pathologists, which can lead to a high rate of false recognition. With the help of machine learning and deep learning knowledge, this infection of leaves can be identified, and take necessary steps in an earlier stage. This will lead to the prevention of the infection rate of other leaves. In this proposed article, three types of soybean diseases such as soybean sudden death, soybean yellow mosaic, and soybean bacterial blight which are significant threats to soybean plant production, have been classified as providing one healthy class.

Strawberries are one of the most sensitive and important crops in the world. Strawberries have high nutritional content and commercial value. So, it is a major fruit for daily consumption Skrovankova et al. (2015). Strawberries are easily infected by several plants’ phytopathogenic fungi, bacteria, and viruses Maas (2012); Pan et al. (2014); Husaini and Neri (2016). That’s why the diseases in strawberry leaves become the main interruption in its yield. Strawberry diseases are manually identified by growers, which is laborious and time-consuming. The shrinking workforce in agricultural counties also complicates this issue, since it is harder to accurately predict disease severity over a large scale. Therefore, it’s urgent to develop an automatic system to identify the diseases in strawberry leaves Xiao et al. (2020). To accomplish the automatic identification of diseases, this article introduces a smart identification system using an image recognition technique for the detection of strawberry diseases using a Convolutional Neural Network (CNN) model. The traditional pathology method involves visually observing diseases, but it is labor-intensive, time-consuming, and heavily dependent on plant pathologists. To address these challenges, the Enzyme-linked Immunosorbent Assay (ELISA) has been suggested, capable of detecting viral protein content in plant extracts Clark and Bar-Joseph (1984). However, it proves less effective for diagnosing fungal and bacterial diseases. Another method, real-time polymerase chain reaction (PCR), is employed for testing plant pathogens, offering superior speed and accuracy compared to the aforementioned techniques Schaad and Frederick (2002). Nevertheless, widespread implementation is hindered by the requirement for skilled operators and the high cost of equipment. Consequently, we propose an image-based diagnostic method using deep learning. This approach is characterized by high accuracy, ease of implementation and the potential for real life implementation. The research offers some contributions. The contributions are –

- Building a deep learning CNN-based model to extract the most relevant features of the plant leaf images.
- Use of machine learning SVM model to classify the diseased and healthy plant leaf images.
- Keeping the model’s parameters low, will produce a low-size model to use comfortably on any device.
- Comparison of the proposed CNN Model with some pre-trained model to show its acceptance and feasibility, as the proposed model is superior to the transfer learning models in terms of parameters and accuracy.
- Comparison with the existing research works by providing the model’s performance in terms of training accuracy, validation accuracy, precision, recall, F1-score, Receiver Operating Characteristics (ROC) curve, precision Vs recall curve, and the number of trainable parameters.
- Use of explainable AI to visualize the diseased areas that classify the plant leaves.

## 2 Related works

The early identification of the plant leaf disease is vital for profitable harvest yield in the agricultural field. Numerous types of research have been carried out to detect the leaf disease on the agricultural land. To achieve this goal, Hang et al. (2019) developed an integrated CNN-based model using squeeze and the Squeeze-and-Excitation module to classify 10 classes of plant leaves for 3 crops - apple, cherry, and corn. To achieve a good classification accuracy and lightweight model, the model was trained using global average pooling layers instead of dense layers. With a dataset containing less number of images, the proposed research work achieved 91.7% accuracy in identifying the diseases in cherries. Zhang et al. (2019) proposed a CNN model which was built based on a pre-trained model named GoogleNet. The model was applied in a binary classification with only 1200 images of cherry plant leaves. The experiment got an accuracy of 99.6% by adopting 5-fold cross-validation.

In order to detect bacteriosis in peach leaves, Akbar et al. (2022) looked for a novel lightweight CNN model based on VGG-19 and got the experimental result with 99% accuracy. The research was a binary classification of healthy and diseased peach leaves with a large dataset. The dataset consists of 1000 images, of which 70% are used for training and 30% for testing the Models. The LWNet Model uses 13 convolutional layers, the count of max-pooling is 7, and the dropout rate is 0.5 with the ReLu activation function. Alosaimi et al. (2021) proposed an innovative method for the binary classification of peach leaves and fruits with 3,199 images. The novel method consists of a CNN-based model and can also locate the region of disease and help farmers find appropriate treatments to protect peach crops. This innovative model got only 94% accuracy.

Soybean is another plant that needs to be identified whether it is infected or not. Wallelign et al. (2018) designed a CNN model based on LeNet architecture to classify four classes including a healthy class of soybean leaf. The authors collected a huge dataset of 12,673 samples and got an impressive accuracy of 99.32%. The research work was classified by only four classes of soybean leaves. Wu et al. (2023) proposed a classification method based on the improved ConvNeXt model where an attention module was used to generate feature maps at various depths and increase the network’s focus on discriminative features as well as reduced background noise. The authors got an experimental accuracy of 85.42% which was comparatively poor in terms of AI-based disease detection. Although the research mentioned some evaluation metrics and a method to visualize the images, the number of model parameters was not satisfactory, as the model was not lightweight. Moreover, the model classified only three classes of soybean leaves including one healthy class. Yu et al. (2022) designed a model by constructing a residual attention layer (RAL) using attention mechanisms and shortcut connections, which further embedded into the residual neural network 18 (ResNet18) model to establish a new model of RANet based on attention mechanism and idea of residuals. The model achieved 98.49% accuracy for the recognition of three types of soybean leaf disease without providing a healthy class. Moreover, their proposed model was not lightweight. Jadhav et al. (2019) presented a novel system using the support vector machine (SVM) and K-Nearest Neighbor (KNN) classifiers used for classifying soybean diseases using color images of diseased leaf samples. The research was applied to the four classes of soybean leaves - blight, brown spot, frog eye leaf spot diseases, and Healthy samples with an accuracy of 87.3% and 83.6%. Besides, the authors didn’t mention the lightweightness of their model and there was no method of visualization through explainable AI in terms of detecting strawberry diseases. The automation of agriculture and image recognition techniques are indispensable.

Xiao et al. (2020) proposed a CNN model based on ResNet50 that achieves a classification accuracy rate of 100% for leaf blight cases affecting the crown, leaf, and fruit; 98% for gray mold cases and 98% for powdery mildew cases. The overall accuracy rate for the feature images of the dataset was 99.60%. The dataset was not augmented as the number of total images was just 1306 and the feature images were built up manually. Moreover, the authors didn’t use some performance evaluation metrics such as confusion metrics, ROC curves, and PR curves to compare the experimental results. Besides, there was no talk about visualization techniques. With the 5 types of classes, the authors managed to get a decent accuracy. Dhivya and Shanmugavadivu (2021) proposed a work that was more concentrated on image pre-processing for the reduction of noise using various filtering methods. The image preprocessing helps to enhance the feature extraction and classification of the leaf disease. The experimental results on the proposed separating model have been assessed regarding PSNR and MSE incentive to clarify and demonstrate the precision of the sifting models by using some image filters based on gradients. Abbas et al. (2021) worked with four pre-trained CNN models to detect the diseases of strawberry scorch with just only 2 types including one healthy class. All the trained CNN models were integrated with a machine vision system for real-time image acquisition. The authors showed an impressive comparison between the transfer learning models and tried to implement the best one for the identification of strawberry disease where EfficientNet-B3 achieved 92% and 97% classification accuracy for initial and severe stage leaf scorch disease respectively. SqueezeNet recorded the lowest disease classification accuracy values in comparison with AlexNet, VGG-16 and EfficientNet-B3. Shoaib et al. (2023) proposed a CNN model that can identify four prevalent diseases: powdery mildew, rust, leaf spot, and blight from 8000 images. The model was trained with multiple hyperparameters, such as the learning rate, number of hidden layers, and dropout rate, and attained a test set accuracy of 95.5%. The authors presented a comparison by changing different hyperparameters and displayed hyperspectral images representing four prevalent types of plant diseases. The results demonstrate that the proposed CNN model performed better when compared with other machine learning image classifiers such as Support Vector Machine (SVM), K-Nearest Neighbors (KNN), Decision Tree, and Random Forest.

Based on the literature reviews, the following gaps have been identified:

- Many studies highlight challenges with limited dataset sizes, impacting the model’s ability to generalize effectively. There is a need for larger and more diverse datasets to enhance model robustness and performance across various environmental conditions.
- The pursuit of lightweight models is emphasized in some studies; however, achieving both high accuracy and model simplicity remains a challenge. Research gaps exist in the development of efficient yet accurate lightweight models suitable for resource-constrained environments, such as on-field applications.
- Several studies achieve high accuracy in disease classification but lack in explaining the affected regions within plant images. Future research should focus on integrating explainable AI techniques to visualize and interpret model decisions, aiding farmers in targeted disease management.
- Some studies fall short in providing a comprehensive set of evaluation metrics, such as confusion matrices, ROC curves, and PR curves. A standardized and thorough evaluation approach is essential for comparing models and understanding their performances.
- Many studies focus on binary or limited multiclass classification, potentially overlooking a broader spectrum of plant diseases. Research gaps exist in addressing challenges associated with an increased number of disease classes and ensuring accurate identification within diverse plant species.
- While several studies propose innovative models, there is often a lack of emphasis on the lightweight nature of these models, critical for practical on-field applications. Future research should prioritize the development of lightweight models without compromising accuracy.
- Certain studies lack comprehensive comparisons between different models or hyperparameters, limiting insights into the effectiveness of various approaches.
- While some studies explore hyperparameters, there was no room for more systematic investigations into the impact of hyperparameter variations on model performance.

With the advancement of machine learning, all the traditional techniques of observing plant diseases have been considered time-consuming and complex. To assist farmers in increasing crop production and identifying diseases at earlier stages, this research proposed a CNN-based technique that combines machine learning and deep learning models. Our research purpose is to make the farmers familiar with the advancement of modern technology easily and identify plant diseases without any confusion. To achieve this goal, different performance evaluation metrics have been added to this research that represent the acceptance of our CNN-SVM model.

## 3 Materials and methods

For the identification of four plant leaf diseases, a 2D CNN-SVM model has been proposed in this research. The model was trained using the Kaggle platform to get the advantages of a Graphics Processing Unit (GPU). To implement the model, various Python libraries like numpy, and pandas and machine learning frameworks like tensorFlow, and keras were applied. Additionally, an explainable AI technique Grad-CAM was used to know the explanation of the outcome performed by the proposed model.

### 3.1 Overall process of establishing the recognition model

Firstly, a large dataset containing ten classes of four types of crop images was collected combined from Kaggle datasets named ‘PlantVillage’ and ‘Soybean Diseased Leaf Dataset’. In the final dataset, we collected four plants (peach, cherry, soybean, and strawberry) healthy and diseased data. After collecting the dataset, we did feature scaling (Normalization) to make our picture size similar and data augmentation like rotating those pictures in different positions to train our model correctly. So, data augmentation is used to increase the diversity and size of a training dataset by applying various transformations to the existing data. By generating new samples from the original data through transformations such as rotation, flipping, cropping, scaling, or adding noise, data augmentation helps improve the robustness and generalization of deep learning models. After data augmentation and scaling, the dataset was ready to be trained by our proposed CNN-SVM model.

**Figure 1:**
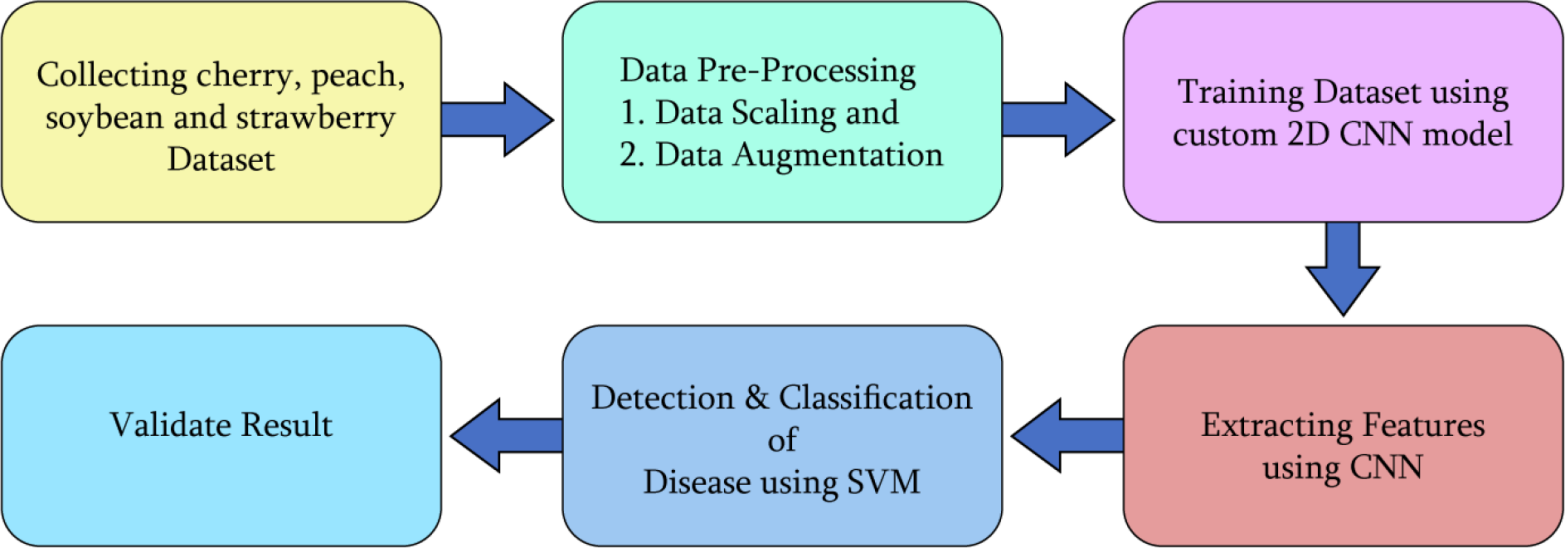
An overview of the whole methodology of the research.

As demonstrated in **Figure 1**, for the identification of four plant leaf diseases, a 2D CNN-SVM model has been proposed in this research. CNN model has the power of extracting features efficiently which helps in the classification system. The CNN model has been fed an enormous dataset that was also augmented to get a generalized and reliable model. In this research, for classification, we used a machine learning model Support Vector Machine (SVM) that works with numerical data. Therefore, CNN works as the collector of featured data for the SVM model. Moreover, Convolutional Neural Networks (CNNs) have revolutionized image analysis and pattern recognition, offering several advantages over traditional observation methods. By implementing the CNN, we extracted features from the dataset, Now, we need to detect and classify key classes from those features in this step we used SVM, a machine learning method for classification. By implementing SVM, we successfully classified the healthy and diseased classes of the cherry, peach, soybean, and strawberry. After correctly classifying the healthy and disease classes, we validate the result by obtaining some performance evaluation metrics - training and validation accuracy curve, loss curve, ROC and confusion matrix.

### 3.2 Dataset description

The importance of a well-curated and representative dataset in deep learning research cannot be overstated. A dataset serves as the foundation upon which deep learning models are built, trained, and evaluated. The quality, diversity, and size of the dataset directly influence the performance, generalization, and reliability of the models developed.

**Figure 2:**
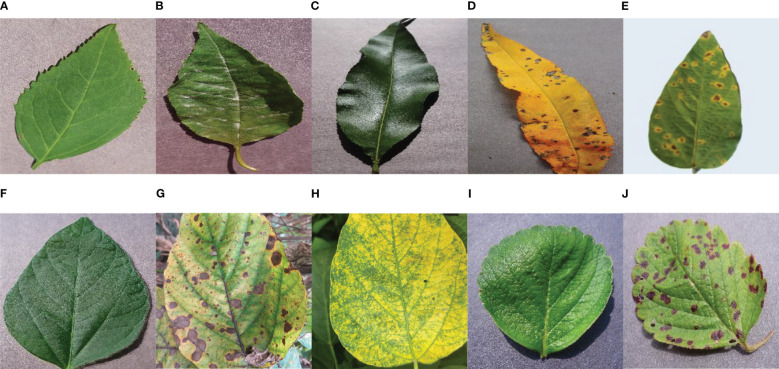
Visual Description of the dataset – **(A)** Cherry Healthy Leaf **(B)** Cherry Mildew **(C)** Peach Healthy Leaf **(D)** Peach Spot **(E)** Soybean Bacterial Blight **(F)** Soybean Healthy **(G)** Soybean Sudden Death **(H)** Soybean Yellow Mosaic **(I)** Strawberry Healthy **(J)** Strawberry Scorch.

To maintain the good performance, generalization, and reliability of the proposed model, a dataset with four types of plant leaves was collected from the publicly available ‘PlantVillage’ dataset and public available Kaggle ‘Soybean Diseased Leaf Dataset’. The following **Table 1** shows that a total 11,504 numbers of plant leaf images were used as the dataset to feed the proposed novel model. The merged dataset consists of four plant leaves – Cherry, Peach, Strawberry, and Soybean. Each type of plant includes healthy and some diseased classes. To make the model well-trained, a total 9,220 numbers of images have been used as training datasets, and 2,304 images for testing purposes are organized into 10 classes (Six diseased classes and four healthy classes). Therefore, the split ratio of the training and testing dataset is approximately 4:1. Here, **Figure 2** depicts example images from all the classes of the dataset.

**Table 1:** Dataset details.

Plant	Disease Type	Training	Testing
Cherry	Cherry Mildew Cherry Healthy	842 682	210 171
Peach	Peach Spot Peach Healthy	1,838 288	459 72
Strawberry	Strawberry Scorch Strawberry Healthy	887 365	222 91
Soybean	Soybean Bacterial Blight Soybean Sudden Death Soybean Yellow Mosaic Soybean Healthy	71 88 88 4,071	17 22 22 1,018
Total		9,220	2,304

### 3.3 Data preprocessing

Image processing plays a pivotal role in enhancing the effectiveness of deep learning models by facilitating the extraction of meaningful features from visual data. In the area of computer vision, where deep learning models are commonly employed for image classification, object detection, and segmentation tasks, raw images often contain an abundance of information. In this research, for the processing of images, two steps have been followed.

#### 3.3.1 Data scaling/resizing

Data scaling or resizing is a crucial preprocessing step in the realm of deep learning, especially for models designed to extract features from diverse datasets. Resizing involves adjusting the dimensions of input data to a uniform size. By bringing input features to a standardized scale, the optimization process becomes more efficient. In this study, the images were resized into 120 X 120 for both the proposed 2D CNN-SVM model and the transfer learning models. Therefore, it becomes ideal to measure the performance of the proposed model and transfer the learning model on a uniform scale.

#### 3.3.2 Image augmentation

**Figure 3:**
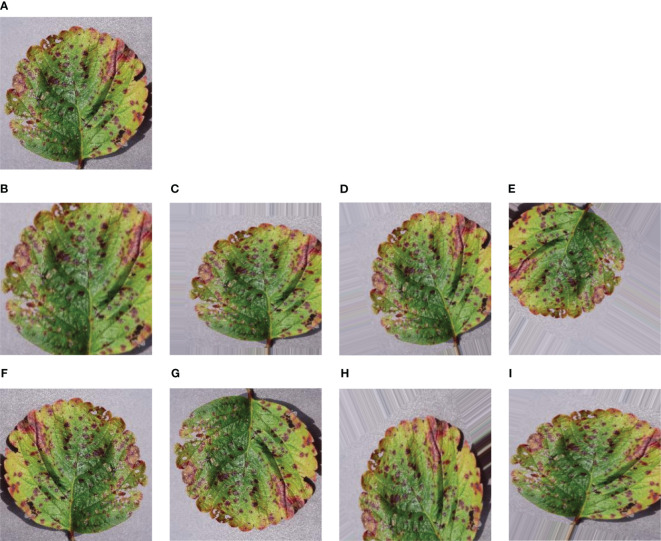
Sample of some augmented leaf images – **(A)** Original leaf image **(B)** Zoomed image **(C)** Sheared image **(D)** Rotated image **(E)** Fill Mode image **(F)** Horizontally Flipped image **(G)** Vertically Flipped image **(H)** Height Shifted image **(I)** Width Shifted image.

Augmentation is a useful technique to make our model more adaptable and avoid getting too focused on specific details. We applied augmentation to generate more images and increase the dataset’s size. The main goal of augmentation is to add some variety to the images quantitatively, which aids the model in avoiding overfitting during training. Overfitting happens when the model starts memorizing random details instead of grasping the actual patterns in the data. Augmentation achieves this by introducing distortions to the images. As demonstrated in **Figure 3**, data augmentation includes different tricks like zooming, shearing, rotating, shifting in height and width, and flipping horizontally or vertically. These techniques create a diverse set of images for our model to learn from, promoting better generalization. For this purpose, some augmentation techniques have been applied in the training images so that the model can observe the dataset from various aspects and validate the dataset from the memorized features. After applying eight techniques of data augmentation, our training dataset gathered a huge collection of datasets. So, a total of 73, 760 images were achieved from the augmentation.

### 3.4 Proposed hybrid method of CNN and SVM

**Figure 4:**
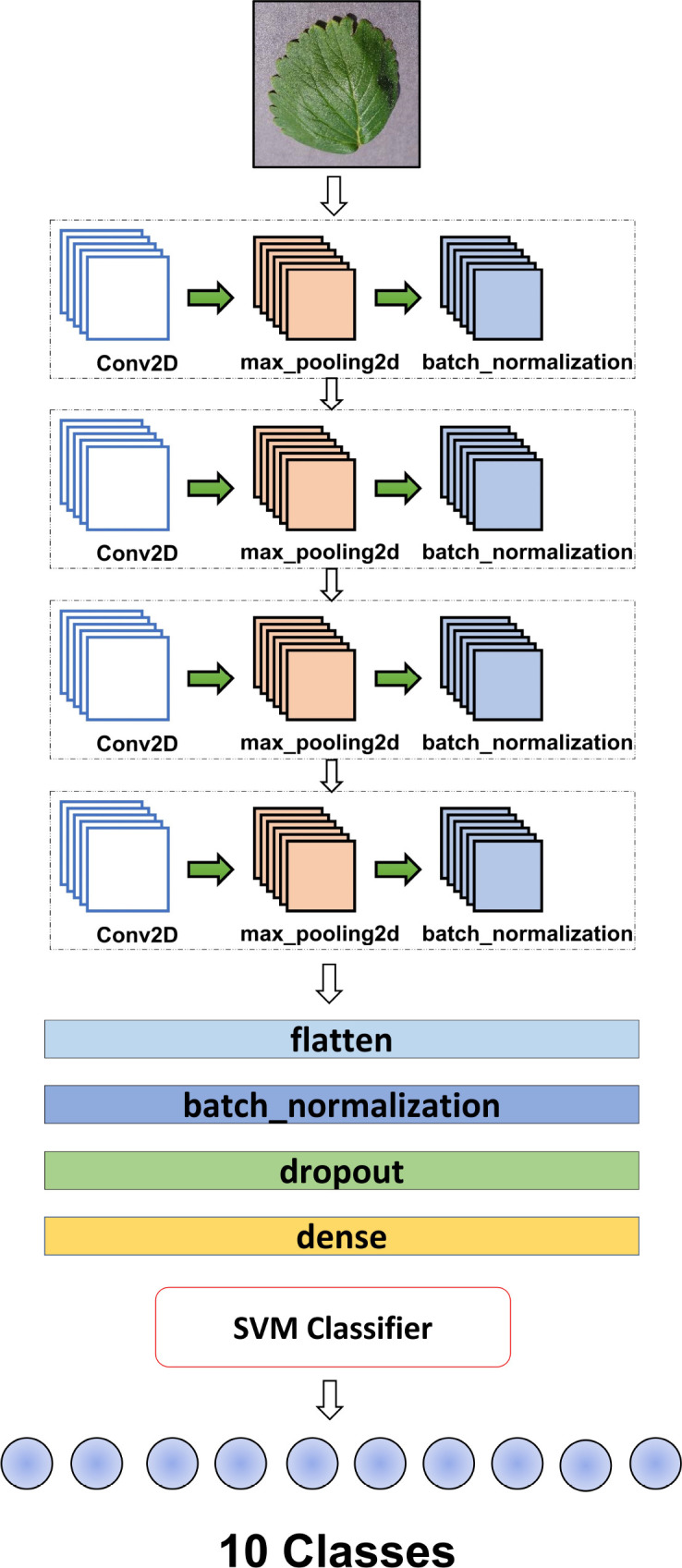
An overview of the whole methodology of the research.

The proposed hybrid (CNN-SVM) model is designed to combine both CNN & SVM advantages for the good classification of plant diseases. In this research, a simple structured 2D CNN model has been proposed to absorb the most important features in the plant leaf images. As CNN is a powerful tool for extracting features and taking two-dimensional inputs, we chose the CNN model to reach our goal. Moreover, enhancing the classification performance of the model relies on extracting distinctive features specific to different leaf diseases. These distinctive attributes play a crucial role in effectively categorizing leaf diseases. The architecture of the suggested 2D CNN model is depicted in **Figure 4**. The model has been formed using four convolutional and max-pooling layers. A max-pooling layer was added following each convolutional layer. Each layer is followed by a batch normalization layer.

The batch normalization layer speeds up the training process of the model. The utilization of batch normalization was implemented to enhance and expedite the model’s performance by readjusting and rescaling the inputs of the layers Santurkar et al. (2018). Besides, the max-pooling layer assumes a pivotal role in the feature extraction process within convolutional neural networks (CNNs). Its primary function involves reducing the spatial dimensions of input feature maps and effectively downsizing them while preserving essential information. This downsampling operation facilitates the identification of prominent features by emphasizing the most significant values within local regions and removing useless data. This process is called subsampling.

In essence, MaxPooling contributes to the extraction of dominant features by highlighting the highest values, resulting in a more refined and condensed representation. Another important step used in the model is to flatten the layer. when the pooling layer is applied and the all-important feature is mapped, the flatten layer converts 2D arrays to 1D arrays before applying a fully connected layer (CNN-SVM) and is followed by batch normalization. In this context, the utilization of dropout aimed to mitigate overfitting by intermittently excluding the training of all nodes within each layer throughout the training process. This strategic approach led to a notable acceleration in training speed, contributing to more efficient model training Peyal et al. (2023). After accelerating the training speed, it is crucial to note that the fully connected layer represents the final layer of a neural network. In all neural networks, every node in this layer is properly connected, and the last layer of the model works as a machine learning classifier named Support Vector Machine (SVM). This layer classifies our research goal using the numerical features collected from the CNN model. This layer ensures that the information learned and processed through the preceding layers is synthesized to produce the final prediction or classification output.

The following **Figure 4** depicts the proposed CNN-SVM model where the CNN model acts as the most relevant feature extractor and the SVM model as the disease classifier. The summary of the proposed model has been drawn in **Table 2**. The table also shows the lightweightness of the model where the number of total parameters is just only 393k which is very impressive and outperforms that of the transfer learning models mentioned in this research. **Table 3** describes all the hyperparameters of the models including 2D CNN-SVM and transfer learning models – VGG16, VGG19, DenseNet, Inception V3, MobileNet, MobileNet V2, ShuffleNet and Xception used in this research.

**Table 2:** Summary of proposed simple 2D CNN model.

Layer (type)	Output Shape	Parameters
L1 (Conv2D)	(None, 120, 120, 16)	448
max_pooling2d (MaxPooling2D)	(None, 60, 60, 16)	0
batch_normalization (Batch Normalization)	(None, 60, 60, 16)	64
L2 (Conv2D)	(None, 60, 60, 32)	4640
max_pooling2d_1 (MaxPooling2D)	(None, 30, 30, 32)	0
batch_normalization_1 (Batch Normalization)	(None, 30, 30, 32)	128
L3 (Conv2D)	(None, 30, 30, 64)	18496
max_pooling2d_2 (MaxPooling2D)	(None, 15, 15, 64)	0
batch_normalization_2 (Batch Normalization)	(None, 15, 15, 64)	256
L4 (Conv2D)	(None, 15, 15, 128)	73856
max_pooling2d_3 (MaxPooling2D)	(None, 8, 8, 128)	0
Flatten (Flatten)	(None, 8192)	0
batch_normalization_4 (Batch Normalization)	(None, 8192)	32768
dropout (Dropout)	(None, 8192)	0
dense (Dense)	(None, 32)	262176
dense_1 (Dense)	(None, 10)	330
Total parameters:		393674
Trainable parameter:		376810
Non-trainable parameter:		16864

**Table 3:** Evaluation metrics comparison with transfer learning models.

Models	Accuracy	Precision	Recall	F1-Score	Parameters	Model Size (MB)
DenseNet	53.82%	76%	78%	70%	7053642	26.91
Inception V3	97.70%	98%	97%	97%	47521706	181.28
MobileNet V2	77.65%	97%	97%	97%	3579978	13.66
MobileNet	69.70%	94%	83%	83%	3250058	12.40
ShuffleNet	98.83%	100%	100%	100%	967874	3.70
VGG 16	98.35%	96%	94%	95%	24683850	94.16
VGG 19	97.61%	97%	96%	96%	20106314	76.70
Xception	84.85%	88%	80%	82%	20881970	79.66
Proposed model	99.09%	99%	99%	99%	393674	1.50

To show the acceptance of the 2D CNN-SVM model, the hyper-parameters were kept the same for the training purpose of all transfer learning models. Overall, the experiment helped to detect the plant leaf diseases impressively.

## 4 Experiment and results

### 4.1 Experimental environment

The experimental environment for image classification using Convolutional Neural Network (CNN) and Support Vector Machine (SVM) involved the utilization of the Kaggle platform, leveraging its available Nvidia P100 GPU with specifications including 16 GB of GPU memory, a clock speed of 1.32 GHz, and a performance capability of 9.3 TFLOPS. To enhance model training efficiency, the input sample size for plant disease images was adjusted to 120 × 120 pixels to match the real-world operating conditions. The training process employed a batch size of 32 for training samples over 350 epochs. The Rectified Linear Unit (ReLU) activation function was applied, and batch normalization was incorporated to normalize batch data. The RMSprop optimizer with a learning rate of 0.001 was chosen for model optimization. Both the proposed CNN-SVM model and transfer learning models shared the same training and validation set sample sizes, training batch configuration, and activation function in the experiment.

### 4.2 Performance metrics

A classification report serves as a comprehensive overview of how well a model performs by highlighting crucial metrics like precision, recall, and F1-score for individual classes. Precision assesses the accuracy of positive predictions, while recall measures the model’s capability to identify all relevant instances. The F1 score combines precision and recall, presenting a consolidated metric. Additional metrics such as accuracy, indicating overall correctness, and the confusion matrix, which breaks down true positives, true negatives, false positives, and false negatives, contribute to a thorough evaluation. Besides the Precission-Recall curve (PR), Region of Convergence (ROC) and loss curve were also used indicating the overall impressive function of the research. These metrics together provide a detailed insight into a model’s strengths and weaknesses, enabling practitioners to make well-informed decisions regarding model improvement and selection based on the specific demands of the image classification task. Thus, the performance of the CNN models was evaluated with these different evaluation metrics. Precision, recall, F1Score, and test accuracy metrics were used to evaluate the performance of the convolutional neural network models that were used in training. Validation and test outcomes for all CNN models were adapted in matrices of binary confusion, which are true positive (TP), false positive (FP), true negative (TN), and false negative (FN) Skrovankova et al. (2015). The first performance evaluation criterion, Accuracy rate, is used to evaluate the performance of network models. The accuracy rate refers to the proportion of the number of corrected positive predictions to that of the whole positive predictions Hang et al. (2019). It signifies the ratio of accurately identified images to the total number of images and is expressed by:


$$\text{Accuracy}=\frac{TP+TN}{TP+TN+FP+FN}$$

Precision measures how accurate your model is when it predicts positive instances. It’s calculated by taking the number of true positive predictions and dividing it by the total number of positive predictions (both true positives and false positives). It can be quantified as,


$$\text{Precision}=\frac{TP}{TP+FP}$$

The Recall measures the efficiency of the neural network in identifying and categorizing the target, determined through the following calculation:


$$\text{Recall}=\frac{TP}{TP+FN}$$

The F1-score serves as the harmonic mean of precision and recall, providing a balanced metric that considers both false positives and false negatives. It is calculated by taking the reciprocal of the average of precision and recall through the following equation:


$$F_{1}-\text{Score}=\frac{2\cdot \text{Precision}\cdot \text{Recall}}{\text{Precision}+\text{Recall}}$$

### 4.3 Multiclass classification results

#### 4.3.1 Accuracy graphs

**Figure 5:**
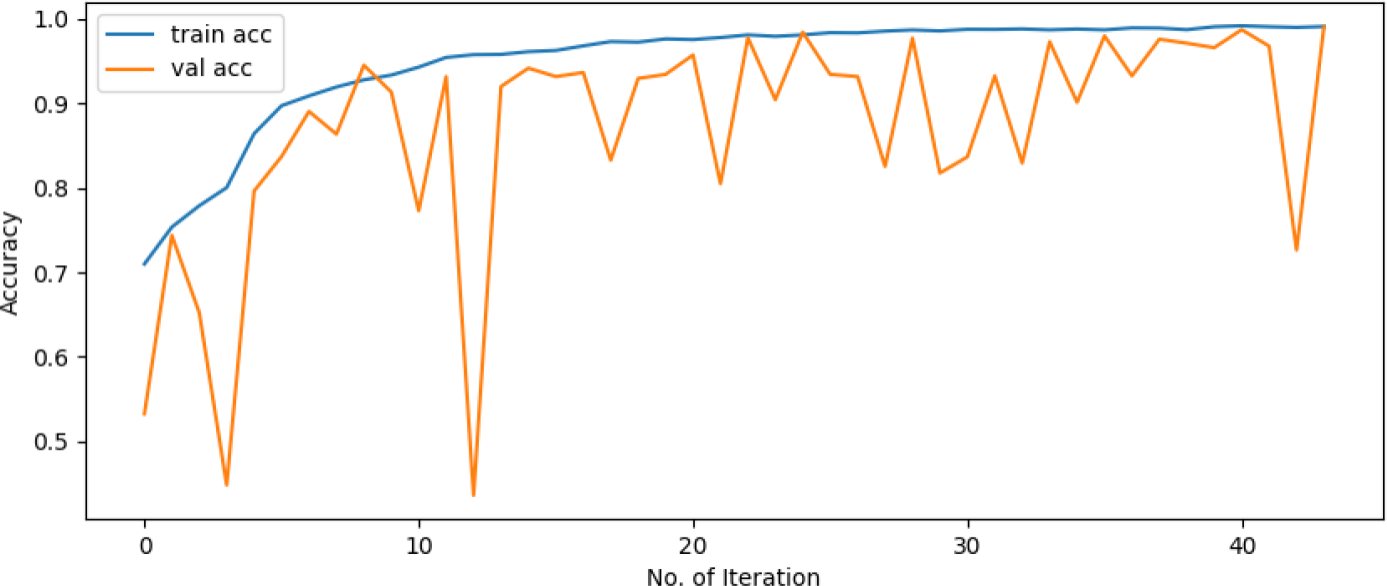
Accuracy graph of the proposed CNN-SVM model.

Accuracy is defined as the sum of correct classifications divided by the total number of classifications. The sum of all diagonal elements is divided by the sum of all items in the confusion metrics. Accuracy gives the overall correctness of the predicted model. The accuracy of the model is drawn across the number of epochs which is called the accuracy graph. The accuracy graph contains both the training and validation accuracy (99.15% and 99.09%) in terms of epoch numbers. According to our research, the first adoption of the proposed CNN-SVM model has been clear from the accuracy graphs of our proposed CNN-SVM model which is shown in **Figure 5**.

**Figure 6:**
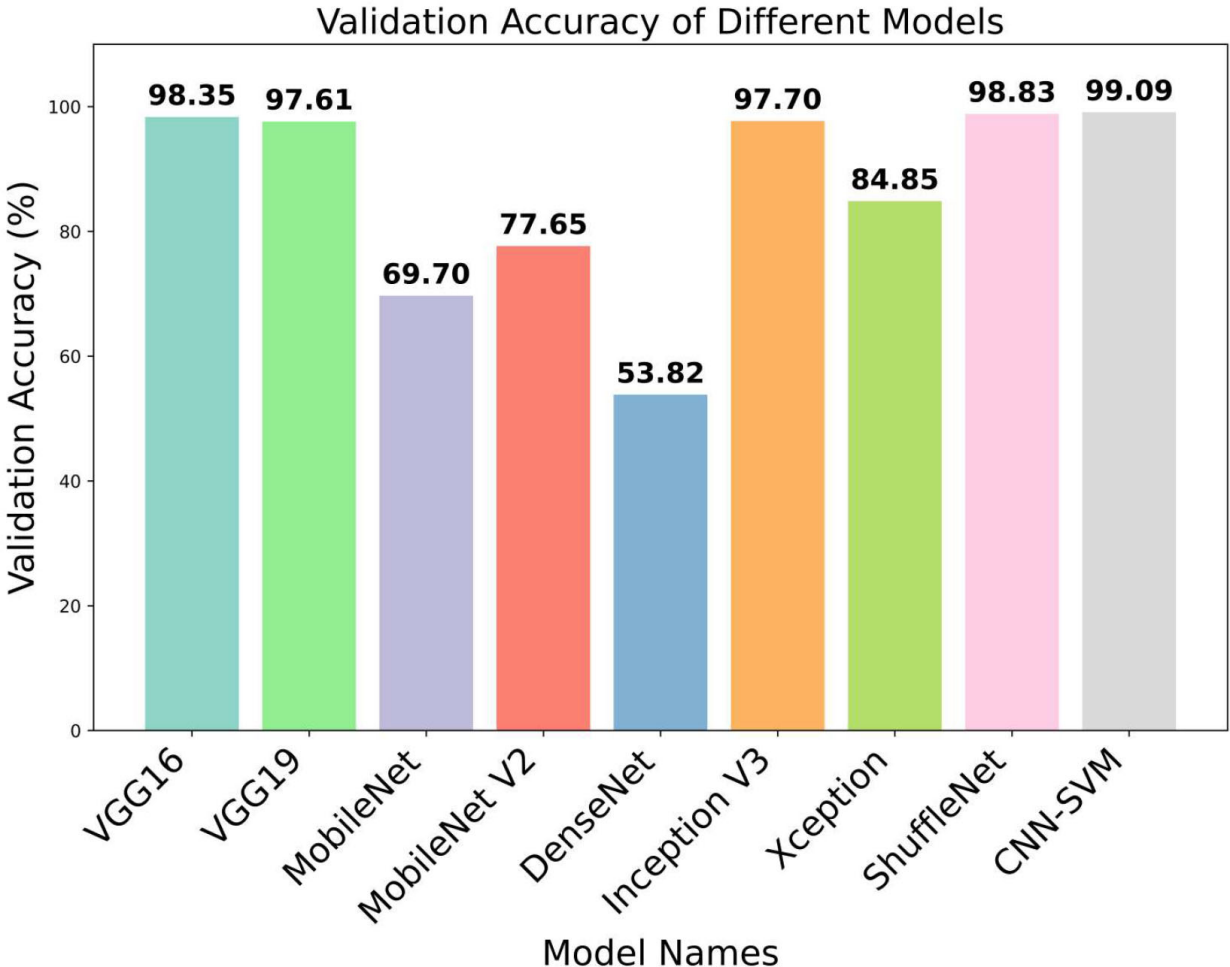
Bar graph of different transfer learning models for validation accuracy.

From **Table 3**, it is observed that the validation accuracy of VGG16, VGG19, Inception V3, shuffleNet, MobileNet, MobileNet V2, DenseNet and Xception are 98.35%, 97.61%, 97.70%, 98.83%, 69.70%, 77,65%, 53.82% and 84.85% respectively. On the other hand, we checked our model in various epochs and environments (**Table 4**) and got the accuracy of 99.09%. In **Figure 6**, the accuracy comparison bar graph has also been shown to observe the outcome of various transfer learning models and the proposed model. Therefore, it is evident that the evaluation metrics accuracy, precision, recall, and F1-score of the proposed model are significantly higher than the transfer learning models which is a very good indicator of the reliability of the proposed model’s performance in classifying 10 categories of plant leaf diseases from a huge dataset using CNN-SVM combined model.

**Table 4:** Accuracy comparison in various epochs.

Epochs	Callback Function	Accuracy
42	Yes	99.09%
100	No	96.79%
200	No	97.98%

#### 4.3.2 Confusion matrix

**Figure 7:**
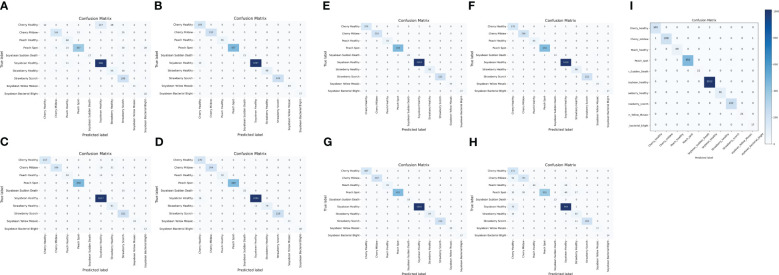
Confusion matrix of **(A)** DenseNet **(B)** Inception-V3 **(C)** MobileNet-V2 **(D)** MobileNet **(E)** ShuffleNet **(F)** VGG16 **(G)** VGG19 **(H)** Xception **(I)** Proposed CNN-SVM model.

The confusion matrix is a table that gives information about how the test dataset performs on the trained model Sharma et al. (2022). Various performance measures like accuracy, precision, recall, or sensitivity and specificity of the model can be calculated using the confusion matrix Tripathy et al. (2015). The diagonal values of the confusion matrix represent true positives (TP). To obtain false negatives, we have to add the values in the corresponding row items ignoring the true positive values. The total number of testing samples belonging to a given class can be calculated by the sum of all items of rows corresponding to that class (TP + FN). Similarly, the number of false positives (FP) for a class is obtained by adding the values of the corresponding column ignoring true positives TP for that class. The total number of true negative TN for a certain class will be the sum of all columns and row values ignoring that class’s column and row. However, this study considered a 10-class problem, which consisted of four healthy classes and six different unhealthy classes of Cherry, Peach, Soybean and strawberry leaves. It is noticeable that out of 2304 images, only 21 images were misclassified by the proposed CNN-SVM model. Therefore, from **Figure 7**, it is clear that the proposed model can classify 10 numbers of classes accurately rather than the existing works.

#### 4.3.3 ROC and PR curves

**Figure 8:**
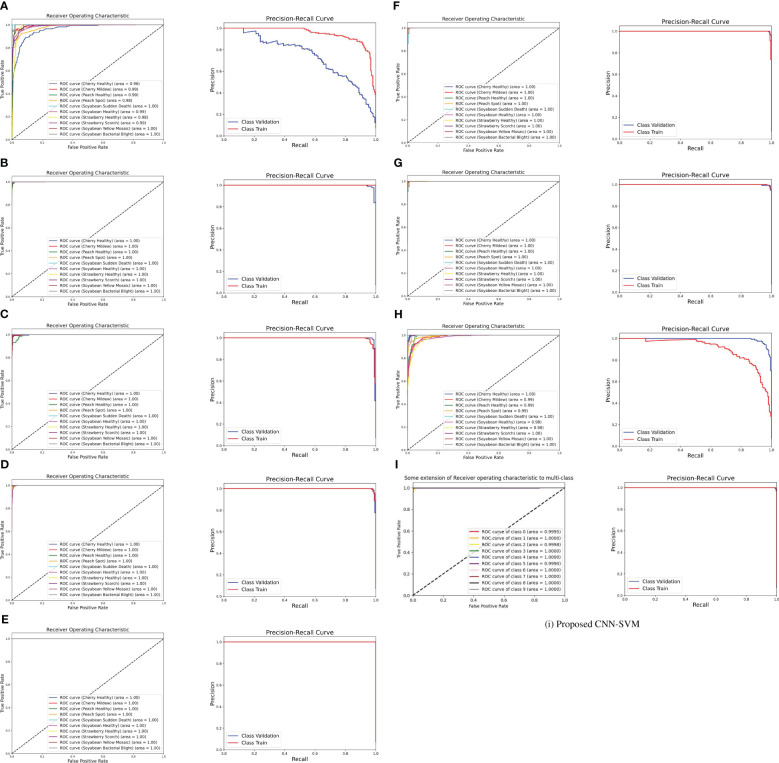
ROC curve and PR curve of **(A)** DenseNet **(B)** Inception-V3 **(C)** MobileNet-V2 **(D)** MobileNet **(E)** ShuffleNet **(F)** VGG16 **(G)** VGG19 **(H)** Xception **(I)** Proposed CNN-SVM model.

The ROC curve is a graphical representation of the trade-off between true positive rate and false positive rate at various thresholds. It is created by plotting the true positive rate against the false positive rate across different classification thresholds. The area under the ROC curve (AUC-ROC) quantifies the overall performance of the model. A higher AUC-ROC indicates better discrimination ability. From **Figure 8**, it is noticeable that the AUC score for the proposed model is almost nearly one and also it has surpassed the other transfer learning model’s AUC. It is also known that a model with a higher AUC-ROC generally performs better. Besides, ROC curves provide insights into the model’s ability to discriminate between classes. On the contrary, The PR curve represents the trade-off between precision and recall at different classification thresholds. Precision is the ratio of true positives to the sum of true positives and false positives. Recall is the ratio of true positives to the sum of true positives and false negatives. From the figure, both the ROC and PR curves show an impressive outcome of the proposed model. In summary, both ROC and PR curves provide valuable insights into different aspects of model performance.

#### 4.3.4 Experimental research from different parameters

In order to achieve a reliable and robust classification model, the research was carried out using different optimizers such as Adam, SGD and RMSprop. The research was done at 350 epochs but we got our expected result within 50 epochs to train the model. In this environment, RMSprop Optimizer has given the best outcome. So, our proposed model gave 99.09% by using RMSprop as an optimizer whereas the SGD and Adam optimizer were not capable of giving this result. The following **Table 5** shows the experimental results in the case of accuracy and AUC score for various optimizers.

**Table 5:** Comparison of various optimizers.

Optimizer	Accuracy	AUC Score
Adam	99%	99.96%
SGD	95%	99.87%
RMSprop	99.09%	99.98%

From the table, it is proved that RMSprop performs better than other optimizers. Overall, the adaptability of RMSprop’s learning rate, its stability during training, efficient memory usage, and rapid convergence made it a favored option across various scenarios, especially when handling complicated deep learning models and extensive datasets.

#### 4.3.5 Matthews correlation coefficient

The MCC is crucial as it considers sensitivity, specificity, precision, and negative predictive value simultaneously, providing a holistic assessment of binary classification models. Matthews Correlation Coefficient (MCC) can also be used in multi-class classification problems but is typically used for binary classification tasks. Unlike the ROC AUC, the MCC generates a high score only when the classifier performs well across all four basic rates of the confusion matrix, ensuring a reliable evaluation.

A high MCC value always corresponds to high values for sensitivity, specificity, precision, and negative predictive value, making it a superior performance indicator compared to other metrics like F1 score and accuracy Chicco and Jurman (2023). The MCC ranges from -1 to +1, with -1 indicating perfect misclassification and +1 indicating perfect classification, while the DOR ranges from 0 to + Chicco et al. (2021).

In our proposed model, We have managed to acquire an impressive outcome of Matthews Correlation Coefficient (MCC) that is 0.987, which signifies a near-perfect classification performance. In summary, the attainment of an MCC value of 0.98 underscores the efficacy and reliability of our model’s classification capabilities. It provides strong evidence that our model has learned meaningful patterns from the data and can generalize well to unseen instances, thereby instilling confidence in its practical utility and real-world deployment.

#### 4.3.6 Mn/Mg deficient leaf vs. soybean sudden death

The symptoms of Mn/Mg deficient leaves and Soybean sudden death leaves are almost similar. These two can look alike, making it hard to distinguish them by eye since they have almost the same features in the images. In this case, we tried to classify them through our proposed model and got an outcome.

To separate the two species through the model, we collected pictures of Mn/Mg-deficient soybean leaves from Google and added those to our dataset after augmenting them.

**Figure 9:**
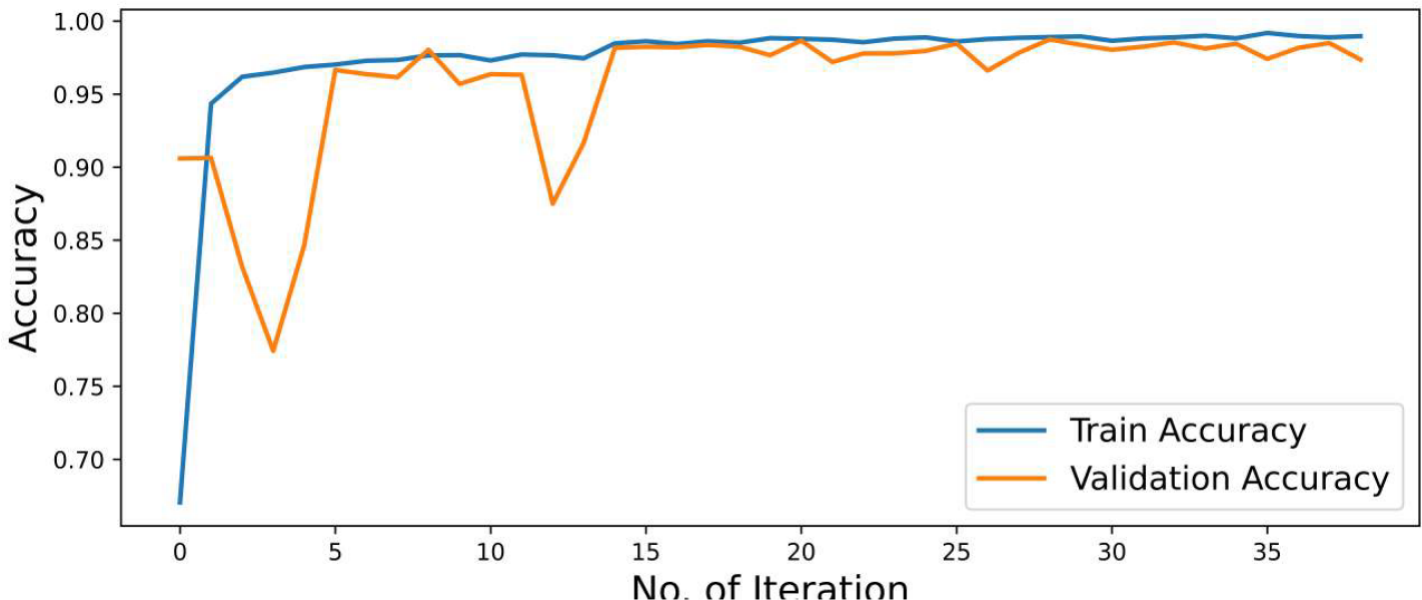
Accuracy graph including Mn/Mg deficiency class.

**Figure 10:**
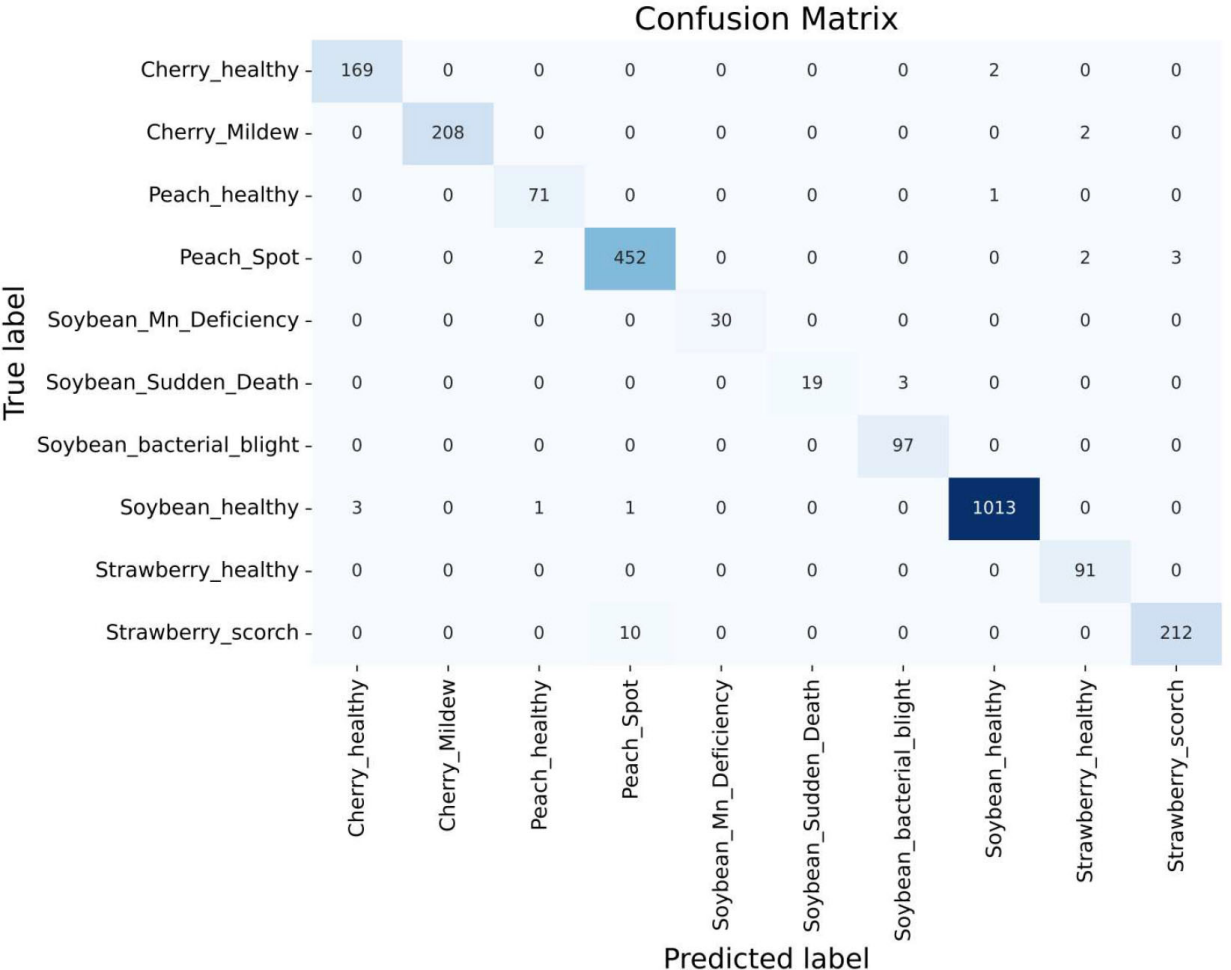
Confusion matrix including Mn/Mg deficiency class.

After adding a new class of Mn/Mg-deficient soybean leaves to our original dataset, the proposed model was applied to the merged dataset. **Figure 9** shows that the model achieved an impressive training accuracy of 99.11% and validation accuracy of 98.74% over the merged dataset. From the **Figure 10** of the confusion matrix, it is seen that our model successfully classified all the images of Mn/Mg deficiency. To make the model recognize the difference among the soybean diseased classes, we increased the number of images in the dataset from ‘DRYAD’ dataset which contains high quality images of the same classes. Eventually, our model became successful in classifying them. In summary, our model has achieved a success rate in distinguishing differences, even when they’re hard to see with the naked eye.

#### 4.3.7 Reusability of the proposed CNN-SVM model

**Figure 11:**
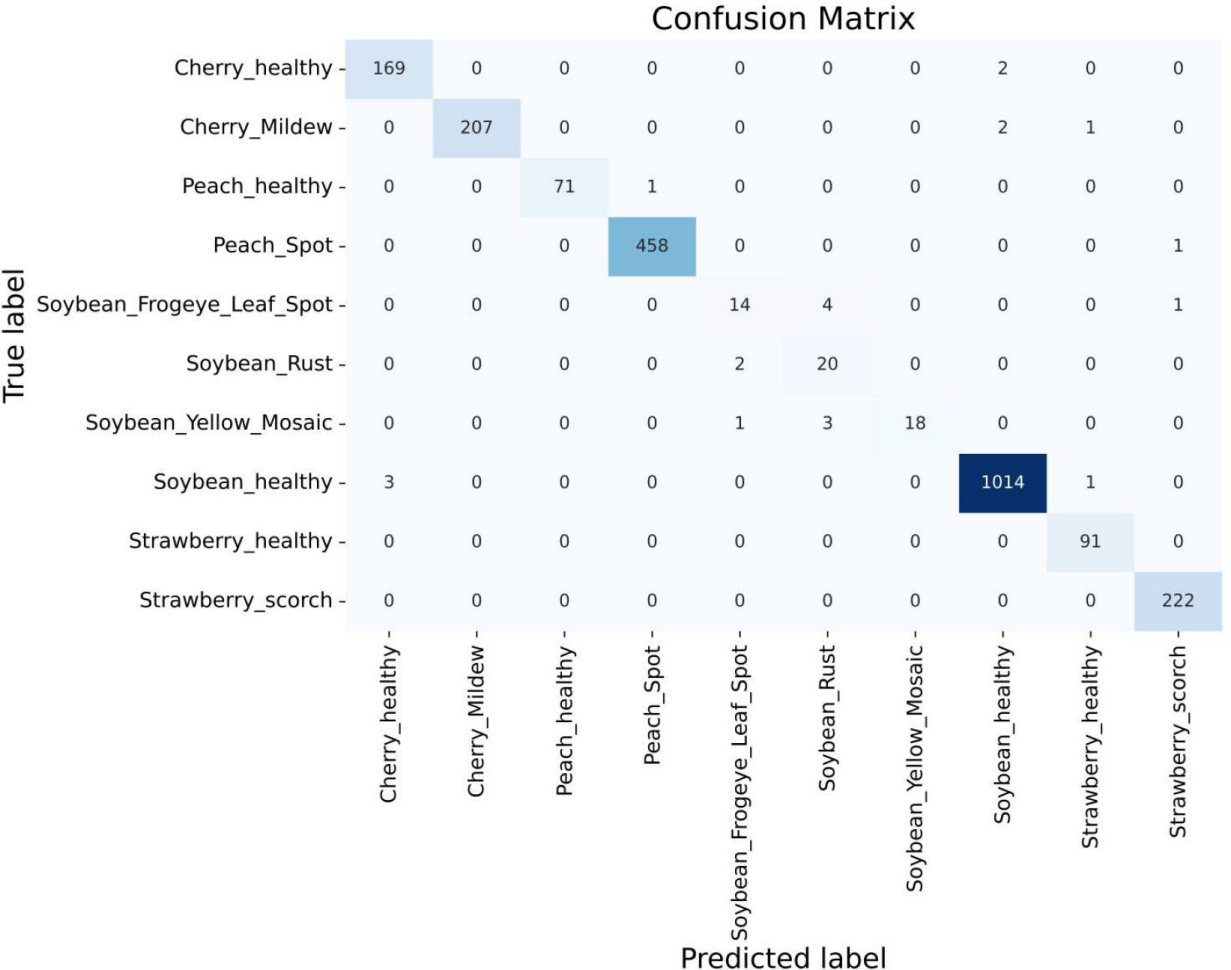
Confusion matrix including soybean frogeye spot and soybean rust classes.

The proposed CNN-SVM model was applied to a new, more extensive dataset comprising larger images of Soybean Rust, Soybean Frogeye Spot, and Soybean Healthy classes, collected from ‘SoyNet’, ‘Soybean Leaf Disease Prediction’, and ‘Roboflow’ datasets. After merging new classes to our proposed dataset, the model was trained on it and achieved an impressive validation accuracy of 99.04%, closely matching our original dataset’s performance. Additionally, as shown in **Figure 11**, the classification for each class was satisfactory like before, maintaining the model’s robust performance.

In **Table 6**, we have evaluated the performance variations of our proposed CNN-SVM model across different criteria. Initially, we observed that certain classes in our proposed dataset contained images that were relatively small in size. To address this, we replaced those classes with new ones featuring comparatively larger images from ‘DRYAD’ dataset which contains great quality images. Additionally, we noted potential confusion between the Mn/Mg deficient class and the Soybean Sudden Death class. To clarify this, we replaced the Soybean Yellow Mosaic class with the Mn/Mg deficient class in our dataset as we wanted to keep the similar types of soybean classes together and reassessed the model’s performance. Finally, we showed the performance of our proposed dataset. Therefore, **Table 6** represents an analysis of using the proposed model across different criteria. This analysis indicates the model’s robustness and effectiveness across various datasets in the desired classification tasks.

**Table 6:** Analysis of applying CNN-SVM over various datasets.

No of Classes	Total Images	Accuracy	MCC
10 (Two new classes - Soybean Rust and Frogeye Spot)	11,532	99.04%	0.98
10 (Replaced Yellow Mosaic with Mn/Mg deficient class)	11,957	98.74%	0.98
10 (Proposed dataset)	11,524	99.09%	0.98

## 5 Comparative analysis

In conclusion, the proposed CNN-SVM model stands as a pioneering solution in the realm of plant disease classification, showcasing a unique fusion of CNN and SVM for optimal feature extraction and classification. The model’s exceptional performance, as evidenced by its accuracy, evaluation metrics, lightweight design, and the incorporation of explainable AI techniques, underscores its superiority. Notably, when compared to well-established transfer learning models such as VGG16, VGG19, MobileNet, MobileNet-V2, DenseNet, Inception-V3, Xception and ShuffleNet, our model emerges as the clear frontrunner. **Table 3** shows that our model performs better than other transfer learning models in terms of accuracy, precision, recall, F1-score, number of parameters and model size. Even when compared to strong competitors like VGG16, VGG19, Inception V3, and ShuffleNet, our model outperforms them across all evaluation measures. Impressively, it achieves superior precision, recall and F1-score metrics, further validating its standard and reliability. Additionally, our model is highly efficient. It’s half the size of the ShuffleNet pre-trained model but still achieves almost similar accuracy. Compared to other transfer learning models, it has the fewest parameters, with some popular models having up to eight times more parameters and larger sizes. This means our model runs fast, making it perfect for various mobile devices. These results suggest that our model is not only effective for diagnosing plant diseases but also has great potential for use by farmers on a large scale. Therefore, its economic feasibility and exceptional performance collectively contribute to its greatness, making it a valuable asset for agricultural practitioners seeking advanced yet accessible solutions.

The proposed CNN-SVM model’s significance is also evaluated against several related research works, where it holds a notable position. Zhang et al. (2019) aimed to develop automatic image-based diagnostic methods for identifying cherry diseases using only two types of cherry leaves – diseased and healthy. The research achieved a high accuracy rate, outperforming other works, and demonstrated its superiority through ROC curves, comparing with various machine learning models. However, they encountered challenges in creating a lightweight model and explaining their model’s visualization technique, such as Grad-CAM. Additionally, they lacked some evaluation metrics like classification reports, confusion matrix, and PR curve. Hang et al. (2019) proposed a model which was compared with numerous transfer learning models regarding accuracy, model size, and training time. Despite having the same number of classes as ours, the paper aimed to structure automatic cherry disease identification with two types of diseased cherry classes and one healthy class. Although the authors visualized the model’s performance, the accuracy rate fell short of expectations and they struggled to develop a lightweight model efficient for farmers.

Alosaimi et al. (2021) showcased impressive results through accuracy graphs, confusion matrices, classification reports and ROC curves, applied to 12 types of peach diseases in a CNN model. They have worked with several peach diseases, but they could also apply their model for the other crops. Besides, their accuracy rate was not as satisfactory as ours and their model lacked visualization technique. Akbar et al. (2022) proposed a novel lightweight and parameters-concerned model for classifying two types of peach leaves, with noticeable experimental outcomes providing various comparisons of performance evaluation metrics and transfer learning models. But, while the accuracy was high, it couldn’t maintain the same accuracy as our proposed model obtained with ten classes. Besides, they could increase the number of peach classes or the types of crops and explain the model by using explainable AI. To sum up, they could increase the dataset by providing more number of classes and trying to achieve the same accuracy as before.

Xiao et al. (2020) proposed research that was conducted with two datasets, utilizing original and feature images to detect strawberry diseases like leaf blight, gray mold, and powdery mildew. Their customized CNN model, based on ResNet50 achieved 99.6% accuracy, but they could have explored more evaluation metrics instead of modifying a transfer learning model. Moreover, they also needed to focus on the number of parameters as ResNet50 has a higher number of parameters. In summary, they have achieved a higher accuracy but with a heavyweight transfer learning model as it has a higher number of parameters. Dhivya and Shanmugavadivu (2021) showed an impressive comparison among various CNN models where EfficientNet-B3 achieved a remarkable outcome than others. However, they haven’t proposed their own built model to compare with various transfer learning models. Moreover, the research paper does not mention the use of visualization techniques like explainable AI and the authors could do the same research for more crops instead of only strawberries. Besides, the authors didn’t show some performance evaluation matrices like the ROC curve, PR curve and Confusion matrix. Another drawback of this research is that the research did not mention the lightweightness of the models.

Wu et al. (2023), the researchers proposed an improved ConvNeXt model with an attention module for generating feature maps at different depths, achieving an accuracy of 85.42% on three types of soybean leaves. Though the number of classes was limited, the accuracy was unsatisfactory, suggesting room for improvement. Jadhav et al. (2019), the authors used SVM and KNN algorithms to classify four types of soybean leaf diseases, achieving 87.3% and 83.6% accuracy, respectively. However, their accuracy value seems to be a limitation due to the use of a small dataset and only one type of crop. Wallelign et al. (2018) managed to achieve 99.32% accuracy with four classes of soybean leaves using a CNN model based on the LeNet architecture, with visualization of the model’s outcome. Although the dataset size was satisfactory, the limited number of disease types was a drawback. Overall, the limitations in existing research, particularly the absence of a combined CNN-SVM model with the Grad-CAM visualization technique have been noticed.

In summary, the discussion highlights our proposed CNN-SVM model having both the advancements and the remaining challenges in automating disease identification in crops. From **Table 7**, our proposed model has mitigated all the research gaps of the existing works mentioned above and showed its acceptance for real-world-based plant disease detection.

**Table 7:** Comparison of existing related works.

Reference	Method	Accuracy	Precision	Recall	F1-Score	Classes	Plant
Zhang et al. (2019)	GoogleNet	99.6%	–	–	–	2	Cherry
Hang et al. (2019)	VGG16	91.7%	–	–	–	10	Apple, Cherry, Corn
Alosaimi et al. (2021)	CNN	94%	94%	94%	94%	12	Peach
Akbar et al. (2022)	LWNet	99%	100%	99%	99%	2	Peach
Xiao et al. (2020)	ResNet50	99.6%	–	–	–	3	Strawberry
Dhivya and Shanmugavadivu (2021)	EfficientNet- B3	97%	98%	97%	97%	2	Strawberry
Wu et al. (2023)	Improved ConvNeXt	85.42%	88.35%	88.44%	88.37%	3	Soybean
Jadhav et al. (2019)	SVM and KNN classifiers	83.6%, 87.3%	–	–	–	4	Soybean
Wallelign et al. (2018)	LeNet	99.32%	99%	99%	99%	4	Soybean
Proposed model	CNN-SVM	99.09%	99%	99%	99%	10	Peach, cherry, soybean, strawberry

## 6 Explainable-AI application

**Figure 12:**
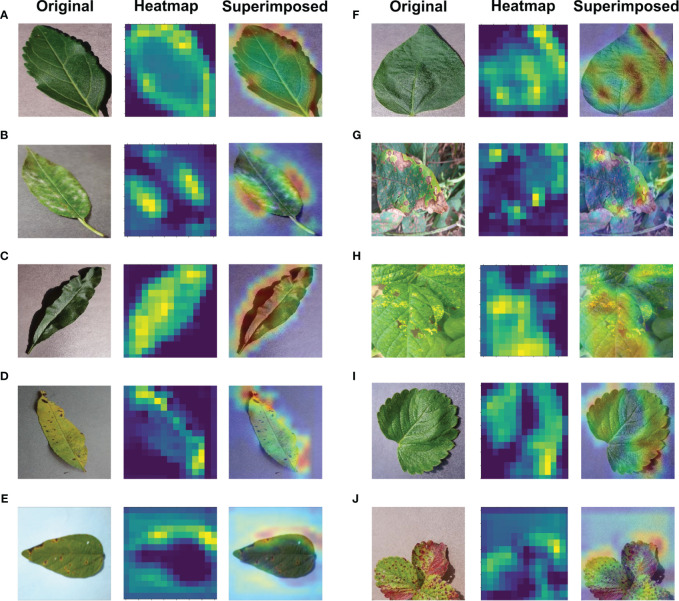
Application of Explainable-AI for **(A)** Cherry Healthy Leaf **(B)** Cherry Mildew **(C)** Peach Healthy Leaf **(D)** Peach Spot **(E)** Soybean Bacterial Blight **(F)** Soybean Healthy **(G)** Soybean Sudden Death **(H)** Soybean Yellow Mosaic **(I)** Strawberry Healthy **(J)** Strawberry Scorch leaves.

Significant efforts are underway to enhance the interpretability and comprehensibility of deep learning, particularly in applications related to the imaging of plant diseases. Ensuring a clear understanding of deep learning models is crucial in such contexts. The Gradient Weighted Class Activation Mapping (Grad-CAM) method, introduced by Selvaraju et al. (2017) plays a pivotal role in elucidating deep learning models as an explainable AI application. Grad-CAM produces a visually interpretable representation of any intricately connected neural network, thereby aiding in model comprehension during task detection or prediction. In the majority of cases, Grad-CAM was primarily applied to the final convolutional layer. Grad-CAM produces a heatmap, highlighting essential areas within an image by leveraging gradients derived from the target class in the last convolutional layer. The regions used for classification become apparent when superimposing this heatmap onto the original image. In this research, Grad-CAM was utilized to asses if leaf sections in the input image significantly influence the diagnostic process to visually depict the diagnosis. The calculation entails evaluating the target class gradient on each feature map and averaging them to determine the relative significance of each map. The computation involves determining a weighted sum of activations from each feature map, where the importance of each is associated with the input image, resulting in the visualization. Grad-CAM proves to be an effective technique that does not hinder performance, as it doesn’t necessitate any additional custom components Fujita et al. (2018). As depicted in **Figure 12**, the proposed model utilized Grad-CAM for detection techniques on a basic image received as input.

## 7 Conclusions

Crop diseases are a major threat to food security, but their rapid identification remains difficult in many parts of the world due to the lack of the necessary infrastructure. The rise in global smartphone usage, along with advancements in computer vision powered by deep learning, has opened doors to smartphone-enabled disease diagnosis. To accomplish this goal, in the proposed work, a 2D CNN-based model has been constructed to detect the 6 disease classes and 4 healthy classes in Peach, Cherry, Soybean, and Strawberry. The suggested 2D CNN-based architecture has four convolutional and four max-pooling layers, two fully connected layers, two dropout layers, and batch normalization in each layer makeup. The suggested model uses less storage capacity and has fewer parameters than transfer learning models because of this kind of shallow structure, which has surpassed heavyweight transfer learning architectures (VGG16, VGG19, and Inception V3) and lightweight transfer learning architectures (MobileNet, MobileNetV2, DenseNet and ShuffleNet) which have an average accuracy range from 54% to 97%. Along with the transfer learning models, the model’s performance has also been evaluated using the confusion matrix, ROC curve, AUC score, and Matthews Correlation Coefficient. The model also showed an impressive performance over various datasets. The outcome shows that the model has achieved a high level of performance that will assist plant doctors and farmers in accurately identifying a variety of diseases affecting cherry, peach, strawberry, and soybean plants. This can help plant doctors take appropriate action to prevent the disease and save money for the farmers. Additionally, this can benefit the economy of the nation. Because the suggested model has significantly fewer parameters than transfer learning models, it requires between three and four times less storage space than transfer learning models. This concept can be easily applied to smartphones and other devices due to its lightweight structure. Grad-CAM class activation maps and a heatmap were created to visualize the detection the trained model was able to achieve to symbolize the area in charge of classification. However, there can be several obstacles and limitations when implementing a model in real-world situations. Besides, our model should have classified Mn/Mg deficient images and Soybean sudden death images without any misclassification although both of the classes have very similar type of features between them. In the future, we have a plan to increase the classification rate more and remove the collision between those two classes. Furthermore, we are planning to explore different hybrid models to handle upcoming challenges better.

## Data availability statement

The original contributions presented in the study are included in the article/**Supplementary Material**. Further inquiries can be directed to the corresponding author.

## Author contributions

RP: Writing – original draft, Software, Methodology, Data curation, Conceptualization, Writing – review & editing, Investigation. AM: Writing – review & editing, Writing – original draft, Methodology, Investigation, Data curation, Conceptualization. HP: Writing – review & editing, Writing – original draft, Software, Methodology, Investigation, Data curation, Conceptualization. SM: Writing – review & editing, Writing – original draft, Methodology, Data curation. MN: Writing – review & editing, Validation, Supervision, Investigation. AK: Writing – review & editing, Validation, Supervision, Project administration, Funding acquisition, Formal analysis. MA: Writing – review & editing, Validation, Supervision, Project administration, Funding acquisition, Formal analysis.
