# Supplementary material for: CSXAI: a lightweight 2D CNN-SVM model for detection and classification of various crop diseases with explainable AI visualization
Source: Front Plant Sci. 2024 Jul 5;15:1412988. doi: 10.3389/fpls.2024.1412988 (PMC11257924; doi:10.3389/fpls.2024.1412988)
Supplement: Supplementary file 1 [file DataSheet_1.pdf]

Supplementary Material

1 SOYBEAN CLASSIFICATION

In order to evaluate our model's performance specifically on soybean data, we have collected 6 more classes of soybean diseased leaves from 'DRYAD' dataset. As our model was trained on cherry, peach and strawberries also, it is expected that the results may differ for 10 classes of soybean leaf images. Besides, the features of cherry, peach and strawberry leaf images on which the model was also trained, are different from the features of the newly added classes of soybean. This can be a vital reason for not getting the accuracy as before. Moreover, we pre-processed the dataset to ensure accuracy, addressing issues like blurriness for optimal results with the 10 soybean classes. Additionally, we resized those images to reduce the computational time of the machine to learn the patterns.

2 SUPPLEMENTARY TABLES AND FIGURES

The model was applied to classify 10 classes of soybean leaf images, achieving a training accuracy of 92.40% and a validation accuracy of 88.33%, as illustrated in Figure S1. Figure S2 illustrates that the model misclassified certain classes to some extent, although the overall classification rate remained higher than the misclassification rate. Figure S3 shows that the model could not perform well for the new classes of soybean. However, the classification rate was somewhat higher than the misclassification rate. Moreover, to achieve a better outcome in the classification of 10 classes of soybean, the model architecture should be modified as the existing model was not trained on the other classes of Soybean.

2.1 Figures

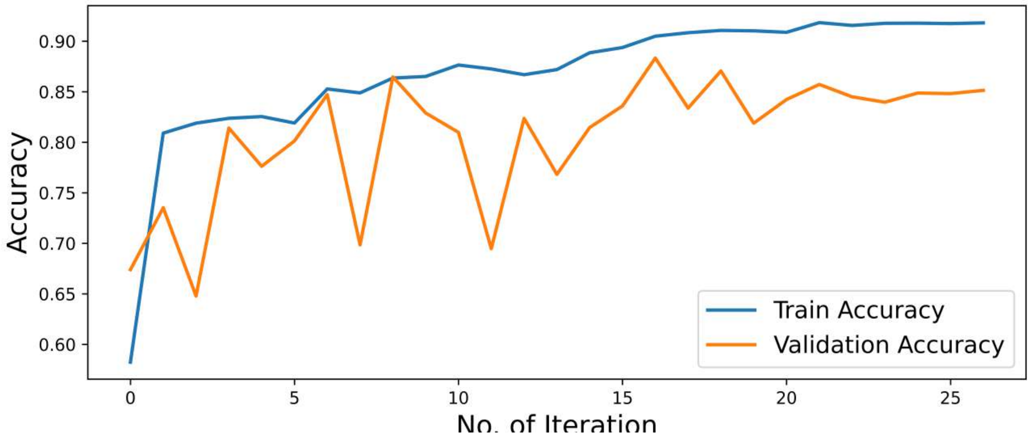

Figure S1: Accuracy graph of CNN-SVM applying on 10 classes of Soybean leaf images

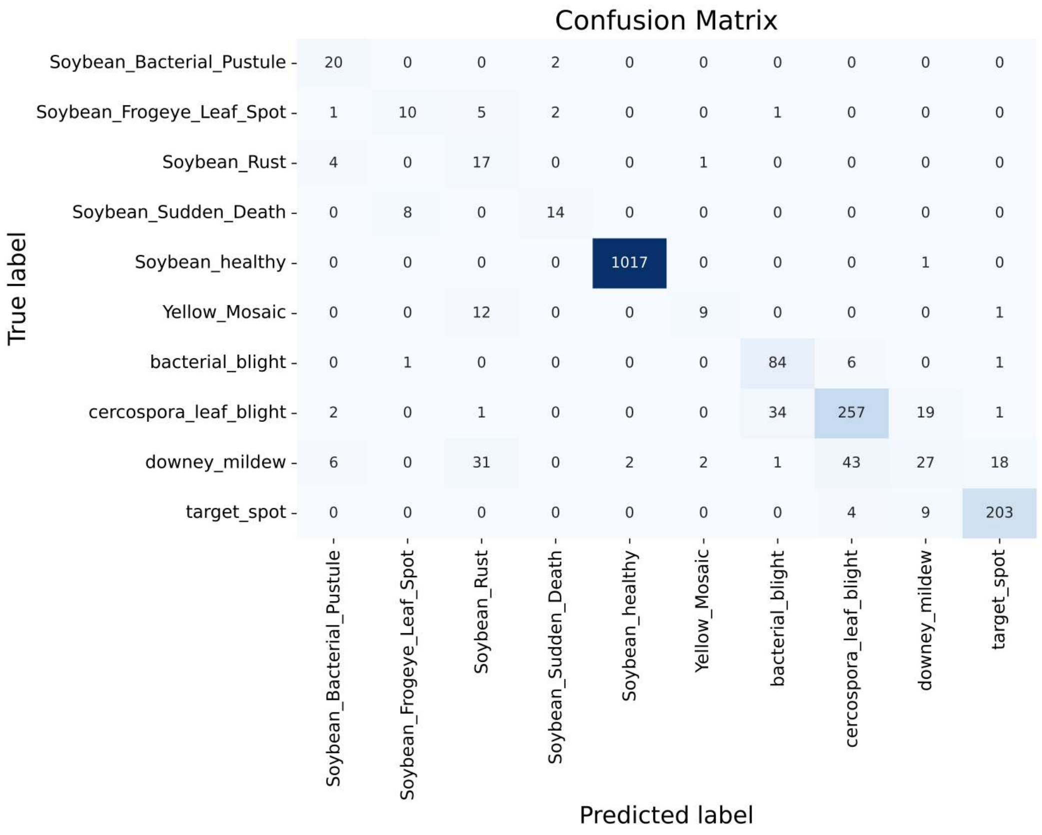

Figure S2: Confusion matrix of CNN-SVM applying on 10 classes of Soybean leaf images

	precision	recall	f1-score	support
Soybean_Bacterial_Pustule	0.61	0.91	0.73	22
Soybean_Frogeye_Leaf_Spot	0.53	0.53	0.53	19
Soybean_Rust	0.26	0.77	0.39	22
Soybean_Sudden_Death	0.78	0.64	0.70	22
Soybean_healthy	1.00	1.00	1.00	1018
Yellow_Mosaic	0.75	0.41	0.53	22
bacterial_blight	0.70	0.91	0.79	92
cercospora_leaf_blight	0.83	0.82	0.82	314
downey_mildew	0.48	0.21	0.29	130
target_spot	0.91	0.94	0.92	216
accuracy			0.88	1877
macro avg	0.68	0.71	0.67	1877
weighted avg	0.89	0.88	0.88	1877

Figure S3: Classification reports for the 10 classes of Soybean
